# Population Differentiation at the PVT1 Gene Locus: Implications for Prostate Cancer

**DOI:** 10.1534/g3.120.401291

**Published:** 2020-05-01

**Authors:** Gargi Pal, Lia Di, Akintunde Orunmuyi, E. Oluwabunmi Olapade-Olaopa, Weigang Qiu, Olorunseun O. Ogunwobi

**Affiliations:** * Department of Biological Sciences, Hunter College of The City University of New York, NY,; ^†^ College of Medicine, University of Ibadan, Nigeria, and; ^‡^ Joan and Sanford I. Weill Department of Medicine, Weill Cornell Medicine, Cornell University, New York, NY

**Keywords:** PVT1 exons 4A and 4B, Black male, prostate cancer, African populations, population differentiation

## Abstract

Genetic variation in susceptibility to complex diseases, such as cancer, is well-established. Enrichment of disease associated alleles in specific populations could have implications for disease incidence and prevalence. Prostate cancer (PCa) is a disease with well-established higher incidence, prevalence, and worse outcomes among men of African ancestry in comparison to other populations. PCa is a multi-factorial, complex disease, but the exact mechanisms for its development and progression are unclear. The gene desert located on chromosome 8q24 is associated with aggressiveness of PCa. Interestingly, the non-protein coding gene locus Plasmacytoma Variant Translocation (PVT1) is present at chromosome 8q24 and is overexpressed in PCa. PVT1 gives rise to multiple transcripts with potentially different molecular and cellular functions. In an analysis of the PVT1 locus using data from the 1000 Genomes Project, we found the chromosomal region spanning PVT1 exons 4A and 4B to be highly differentiated between African and non-African populations. We further investigated levels of gene expression of PVT1 exons 4A and 4B and observed significant overexpression of these exons in PCa tissues relative to benign prostatic hyperplasia and to normal prostate tissues obtained from men of African ancestry. These results indicate that PVT1 exons 4A and 4B may have clinical implications in PCa a conclusion supported by the observation that transient and stable overexpression of PVT1 exons 4A and 4B significantly induce greater prostate epithelial cell migration and proliferation. We anticipate that further exploration of the role of PVT1 exons 4A and 4B may lead to the development of diagnostic, therapeutic, and other clinical applications in PCa.

Cross-population comparisons of health outcome measures, such as disease incidence and prevalence, has contributed to progress in public health research (Moreno-Betancur *et al.* 2018). Multiple studies have assessed the contribution of risk factors specific to geographical areas, ethnicity, and several other factors in different diseases (Babu *et al.* 2012; Krämer *et al.* 2015). Genome wide association studies (GWAS) have identified several single nucleotide polymorphisms (SNPs) associated with different complex diseases (Giri *et al.* 2016; Foley *et al.* 2017; Sud *et al.* 2017). In prostate cancer (PCa), ethnicity, advanced age, and genetic history are considered important risk factors (Chang *et al.* 2018). Other risk factors such as socio-economic status, lifestyle, and environmental factors are also well documented in PCa (Hayes and Bornman 2018). The 8q24 region harbors multiple risk variants for distinct cancers (Han *et al.* 2016). The replication of the association of chromosome 8q24 variants with increased PCa risk in African populations indicates the possible role of this genomic region in the higher burden of PCa in men of African ancestry (Okobia *et al.* 2011; Adeloye *et al.* 2016). It is also found that the incidence and mortality rates are 1.6 - and 2.4 - fold greater for African Americans than for European Americans (Hayes and Bornman 2018). In the USA, the incidence of PCa observed in American Indian/Alaska natives is 46.9 per 100,000, in Asian/Pacific Islanders is 52.4 per 100,000, and in Whites is 93.9 per 100,000. Strikingly, the highest incidence in the USA is seen in African-American men (157.6 per 100,000) (Rawla 2019).

PCa is the most commonly diagnosed solid organ non-skin cancer in males worldwide and the second most common cause of cancer mortality in the United States (Torre *et al.* 2015; Hassanipour-Azgomi *et al.* 2016). In 2015, PCa showed the highest incidence for men in 103 countries or territories (Global Burden of Disease Cancer Collaboration *et al.* 2017). In 2017, there were 1.3 million incident cases of prostate cancer and 416 000 deaths (Global Burden of Disease Cancer Collaboration *et al.* 2019). There are more than 3.1 million American men living with the disease and in 2020, there will be 191,930 new cases of PCa with about 33,330 deaths in the United States (Lewis *et al.* 2017; Viale 2020).

PCa has become the leading cause of cancer-related death in men with increased morbidity in the developing world (Siegel *et al.* 2014). The incidence of PCa is almost 60% higher in men of African ancestry and the mortality rate is two to three times greater than among Caucasian men. Surprisingly, these numbers have remained remarkably constant for more than 20 years (Odedina *et al.* 2009). African American men have among the highest incidence of PCa worldwide, are more likely to develop PCa at any age, and develop the disease earlier in life than men from all other racial and ethnic groups (Kheirandish and Chinegwundoh 2011).

Long non-coding RNAs (lncRNAs) are a class of RNAs longer than 200 nucleotides, which do not encode for functional proteins but play an important role in the regulation of different biological processes (Kung *et al.* 2013). Plasmacytoma Variant Translocation 1 (PVT1) is a long non-protein-coding gene, located at chromosome 8q24 and contains approximately twelve exons and encodes a cluster of six microRNAs (Ilboudo *et al.* 2015; Colombo *et al.* 2015). Several studies have reported that PVT1 plays a role in tumorigenesis, proliferation, apoptosis, cell cycle progression, migration, and invasion (Chen *et al.* 2018a; Derderian *et al.* 2019; He *et al.* 2019; Pal *et al.* 2019; Onagoruwa *et al.* 2020). Aberrations of PVT1 are associated with different malignancies including cervical cancer, bladder cancer, colorectal cancer, gastric cancer, breast cancer, hepatocellular carcinoma, lung cancer, and breast cancer (Wan *et al.* 2016; Iden *et al.* 2016; Chen *et al.* 2018b; Tang *et al.* 2018; He *et al.* 2019; Yu *et al.* 2019). Furthermore, PVT1 is found to be dysregulated in acute myeloid leukemia and Hodgkin’s lymphoma, vitiligo, and asthma (Zeng *et al.* 2015; Austin *et al.* 2017; Ben *et al.* 2018; Chen *et al.* 2018b).

In PCa, PVT1 amplification is correlated with its incidence (Chang *et al.* 2018; Wan *et al.* 2018). PVT1 promotes proliferation, invasion, and metastasis, and promotes epithelial to mesenchymal transition in PCa (Chang *et al.* 2018). Though the role of PVT1 is well-established in different cancers and other diseases, very few reports are available on the alternatively spliced transcripts of PVT1 (Guo *et al.* 2017, Pal and Ogunwobi 2019). We previously demonstrated that PVT1 exon 9 is associated with aggressive PCa in men of African ancestry (Ilboudo *et al.* 2015).

In this study, we sought to uncover if there are any population-level genetic differences in PVT1 and to explore any potential implications for PCa. We performed population analysis and interestingly, PVT1 exons 4A and 4B consistently showed the highest level of genetic differentiation (*F_st_* ∼0.25) between African and non-African populations. We also assessed its expression in histologically confirmed normal prostate, benign prostatic hyperplasia, and PCa tissues. Our results confirmed significant overexpression of PVT1 exons 4A and 4B in PCa tissues in comparison to normal prostate tissue and benign prostatic hyperplasia. Furthermore, both transient and stable overexpression of PVT1 exons 4A and 4B in a non-tumorigenic prostate epithelial cell line (RWPE1) induced increased cell proliferation and migration, which are among the hallmarks of cancer. Notably, stable sublines of the non-tumorigenic prostate epithelial cell lines overexpressing PVT1 exons 4A and 4B demonstrate increased cell proliferation and migration. Here, our results show that overexpression of PVT1 exons 4A and 4B is characteristic of PCa in men of African ancestry. These data suggest that PVT1 exons 4A and 4B may have clinical applications for PCa in men of African ancestry.

## Materials And Methods

### Population-level genetic analysis

We downloaded the Phase 3 release of the variant call file (VCF) on the human chromosome 8 (Build GRCh38, the NCBI Nucleotide accession “NC_000008”) generated by the 1000 Genomes Project (The 1000 Genomes Project Consortium 2015). We used the VCFtools (Danecek *et al.* 2011) to extract SNPs (N = 6,688) within the PVT1 locus using coordinates from 127,890,628 to 128,101,253 according to the December 2013 annotation of the UCSC Human Genome Browser. Note that this stretch of 210.6 kb is shorter than but encompassed by the presently annotated PVT1 locus, which includes 386.6 kb between coordinates 127,794,526 and 128,181,101 on chromosome 8 according to the Ensembl Gene accession “ENSG00000249859”, 306.7 kb between 127,794,533 and 128,101,253 according to the NCBI Gene accession “5820”, or 305.5 kb between 127,795,799 and 128,101,256 according to USCS Human Genome Browser. We calculated genetic differentiation between populations (*F_st_*), nucleotide diversity (*π*), and a measure of natural selection (Tajima’s *D*) using VCFtools (Danecek *et al.* 2011). A custom webpage (http://diverge.hunter.cuny.edu/∼weigang/oneKGenome/) was developed to allow interactive visualization of genetic variations at the PVT1 locus. Haplotypes within the 4A and 4B region, consisting of sequences at the 75 SNP sites within a 11kb region between cooridnates 127,982,050 and 127,992,931, were extracted using a custom PERL script. GenBank SNP database accessions of these 75 SNPs and allele frequencies at individual SNP positions are included in Supplemental Table 1. Haplotype networks were reconstructed using the TCS algorithm and its associated software tool (Clement *et al.* 2000). TCS infers a gene geneaology as well as relative ages of haplotypes based on maximum parsimony probabilities while allowing for recombination. The resulting haplotype networks were rendered using tcsBU (Múrias dos Santos *et al.* 2016). The VCF files, computational scripts (written in BASH, PERL, and R), and output files are available at a GitHub repository (https://github.com/weigangq/pvt1).

### Tissue analysis

Normal prostate tissue (n = 22), benign prostatic hyperplasia (n = 35), and PCa tissue (n = 28) samples were obtained from patients who had undergone prostatectomy or transrectal ultrasound-guided biopsy at the University College Hospital, Ibadan, Nigeria. Tissues were collected in compliance with Institutional Review Board approved protocols and histopathological analysis was performed.

### Cell culture

The prostate epithelial cell line, RWPE1, was cultured in keratinocyte serum-free medium (SFM) supplemented with 0.05 mg/ml bovine pituitary extract (BPE), 5 ng/ml epidermal growth factor (EGF) and 1% penicillin/ streptomycin (P/S).

### Cloning of PVT1 exons 4A and 4B, and establishment of novel stable cell lines overexpressing PVT1 exons 4A and 4B

The PVT1 exons 4A and 4B fragments were synthesized by IDT (USA) and the dsDNA was reconstituted and amplified with polymerase chain reaction (PCR). The forward primers of each set contained HindIII, and reverse primers contained BamHI restriction site. The PCR was performed as follows: initial denaturation of DNA at 98° for 30 sec, an amplification program consisting of 30 cycles at 98° for 10 sec, 55° for 30 sec, and 72° for 30 sec and the final extension of 1 cycle at 72° for 10 min. PCR products were resolved by gel electrophoresis on a 1.8% agarose gel in Tris–acetate buffer (1X). The PCR products were purified with a gel purification kit (Qiagen, Hilbert, Germany) and digested with HindIII and BamHI restriction endonucleases. The resulted gene fragments were purified and ligated into pcDNA3.1(+) vector (Addgene, USA) digested with the same enzymes. Ligation mixtures were transformed into *E. coli* JM109 competent cells, using standard procedures as described by Sambrook *et al.* 2001 (Elder 1983; Maniatis *et al.* 1985). The recombinant plasmids were confirmed by restriction digestion by HindIII and BamHI, colony PCR as well as by sequencing. For stable cell line selection, prostate epithelial cell line (RWPE1) transfected with PVT1 exons 4A, 4B or empty pcDNA3.1 vector was grown in the presence of geneticin (Gibco) at a concentration of 100 μg/ml for two weeks.

### Transfections

RWPE1 cells were seeded in 6-well plates. To investigate the role of PVT1 exons 4A and 4B, the transcripts from PVT1 exons 4A and 4B were cloned into the mammalian expression vector pcDNA3.1 (Invitrogen, Carlsbad, CA, USA). After reaching 70% confluence, media was replaced with Opti-MEM (Thermo Fisher Scientific Inc; Wilmington, DE, U.S.A) and cells were transfected with 100 ng of plasmid construct using Lipofectamine 3000 (Thermo Fisher Scientific Inc; Wilmington, DE, USA), according to the manufacturer’s instructions. Transfected cells were then incubated at 37° for 24 hr prior to replacing with cell line-specific fresh culture media.

### RNA extractions

At 75% confluency, total RNA was extracted from non-transfected and transfected RWPE1 cells grown in 60 X 15 mm tissue culture dishes, using RNeasy Mini Kit (Qiagen, Germany, cat# 74104). After quantification with Nanodrop1000 spectrophotometer (NanoDrop, Madison, WI, USA), 1 μg of RNA was reverse-transcribed into cDNA using QuantiTect Reverse Transcription kit (Qiagen, Germany, cat# 205311).

### Quantitative reverse transcriptase polymerase chain reaction (qPCR)

The qPCR assays were performed on an ABI 7500 platform (Applied Biosystems instruments, Grand Island, NY, USA) with 25 μl reaction volumes containing 12.5 μl SYBR Green PCR master mix (Life Technologies, Grand Island, NY, USA cat# 4309155), 0.4 µM final concentration for primers, 2.5 μl cDNA template, and 7.5 μl of water. The thermal cycle protocol used was as follows: 50° for 2 min, 10 min initial denaturation at 95°, and 40 cycles of 15s denaturation at 94°, 1 min annealing at 58°. A dissociation curve was also added at the end of the cycle.

### Migration assays

Wound healing migration assays were performed as previously described (Ogunwobi and Liu 2011). 10^5^ cells were seeded into 6-well plates. At 90% confluency, the cell monolayer was wounded with a 200 μl-pipette tip, washed with PBS and cell culture medium was replaced. Images were taken at 0 h, 24 h, 48 h, and 72 h intervals. Images were taken using Motic Images Plus 2.0 Software (Motic; British Columbia, Canada).

### Cell proliferation assays

10^4^ cells were seeded into 96 well plates. At 60–70% confluency, the cells were transfected with PVT1 exons 4A, 4B or empty vector. After 24 h, MTT cell proliferation assays were performed and absorbance measured at 490 nm with a microplate reader (Spectramax i3 multimode microplate reader, USA).

### Statistical analysis

The relative mean expression of PVT1 exons 4A and 4B obtained from three different types of tissue samples, namely normal prostate tissue, benign prostatic hyperplasia tissue, and prostate cancer tissue, were used for analysis. For comparison of these three groups, analysis of variance (ANOVA) test was used to check overall statistically significant difference (*P* < 0.05) in group means. ANOVA was performed using the SPSS Statistics software (http://www-01.ibm.com/software/analytics/spss/) on normalized data. As ANOVA result does not identify which particular differences between pairs of means are significant, in the next step, a *post hoc* tukey test was run to analyze the differences between multiple group means while controlling the experiment wise error rate that occurred between the groups. To check PVT1 exons 4A and 4B gene expression in the cell lines used, and the role of PVT1 exons 4A and 4B gene expression in cell proliferation, and migration, qPCR, MTT assay, and wound healing assays were performed. Each of these experimental data were collected from at least three independent biological experiments.The results are presented as the mean± SEM (SEM). The p values were calculated in Microsoft Excel and *P* < 0.05 were considered significant.

### Data availability

Supplementary Figure 1 contains data indicating that transient overexpression of PVT1 exons 4A and 4B promotes cell proliferation and migration. The human tissue study is in compliance with City University of New York approved IRB protocol number 2016-0368. VCF files, VCF tools commands, and computational scripts (in BASH, PERL, and R) are available at a github repository (https://github.com/weigangq/pvt1). Supplemental material available at figshare: https://doi.org/10.25387/g3.9911714.

## Results

### Population-level differences in PVT1

**Figure 1:**
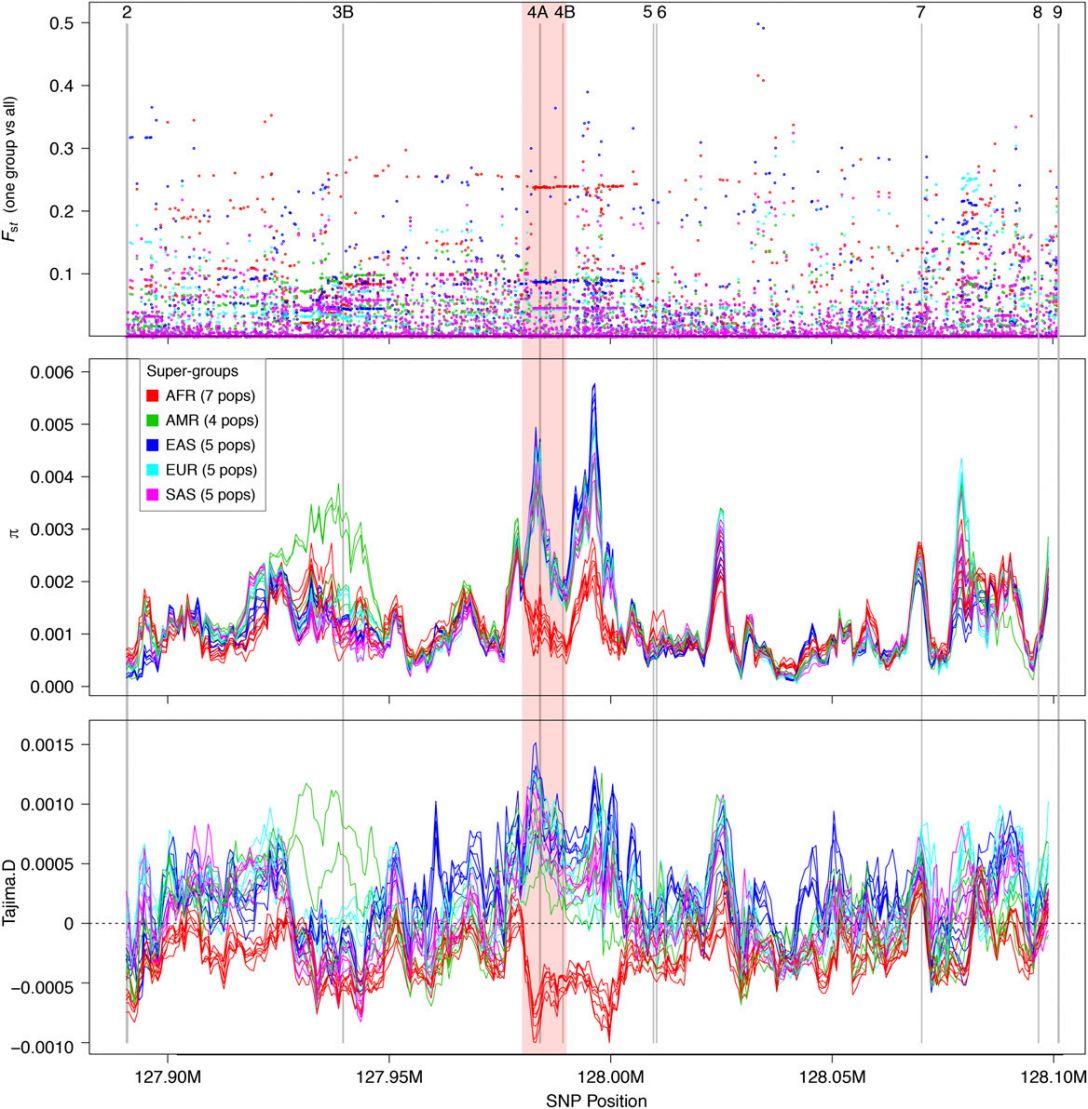
(A) Levels of genetic differentiation (*F_st_*, *y*-axis) at n = 6,688SNP sites between each of the five population supergroups and the combined population. The *x*-axis shows genome coordinates of chromosome 8 (GenBank accession NC_000008), which spans the 211 kb region of the PVT1 locus. A 11-kb region across PVT1 exons 4A and 4B shows a run of 75 SNPs with high *F_st_* values between African populations (in red) and other populations. (B) Nucleotide diversity (*π*) within each of the 26 populations (window size = 100 SNPs, window step = 20 SNPs) (C) Signature of positive natural selection (D > 0) in the 11-kb region in non-African populations and negative selection (D < 0) in African populations.

**Figure 2:**
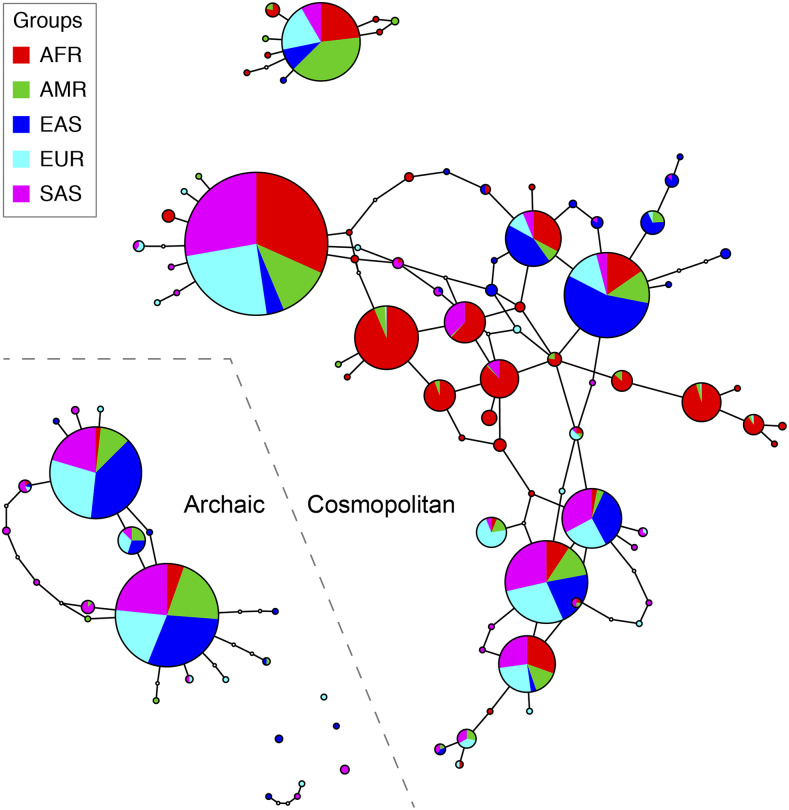
Haplotype networks of sequences at the 75 SNP sites within the 11-kb PVT1 exon 4A/4B region. Each circle represents a unique haplotype. A total of 99 distinct haplotypes were found among 5,008 chromosomes from 2,554 individuals. Sizes of circles are proportional to haplotype frequencies, while the enclosed pie charts show relative frequencies of a haplotype in five population super-groups. Edges linking the circles represent highly probable (with 95% or higher confidence) mutational steps. Disjoint circles indicate a lack of high-confidence mutational links to other haplotypes due to, *e.g.*,an absence of intermedidate haplotypes in the sampled individuals. Relative positions of haplotypes are indicative of their relative ages, with ancestral haplotypes located near the center of a sub-network (*e.g.*, the predominantly African haplotypes in the midde of the largest sub-network) and recently evolved haplotypes located on the peripherals of a sub-network. Cyclic sub-networks indicate presence of recombination while acyclic sub-networks indicate mutation-only changes.The bottom-left subnetworks consist of predominantly non-African haplotypes presumably due to intragression from archaic humans (“archaic” haplotypes). The remaining haplotype networks consist of haplotypes present in all five super-groups (“cosmopolitan haplotypes”). Haplotypes present in high frequencies in African populations (*e.g.*, those at the center of the largest network) and haplotypes nearly absent in African populations (“archaic” haplotypes) are both candidate genetic risk factors for PCa.

To identify racial differences in the PVT1 locus, we scanned for signatures of population differentiation and positive natural selection using the latest (Release GRCh38) full-genome variability panel from the 1000 Genomes Project. A string of 75 SNPs in an11-kb region spanning PVT1 exons 4A and 4B consistently show the highest level of genetic differentiation (*F_st_* ∼0.25) between African and non-African populations (Figure 1A). The 11-kb region shows the highest levels of sequence diversity in non-African populations as well (Figure 1B). Tajima’s D statistic shows the signature of positive natural selection (D > 0) in the 11-kb region in non-African populations and negative selection (D < 0) in African populations (Figure 1C). The 99 distinct 4A/ 4B haplotypes (sequences at the 75 SNP sites within the 11-kb region) found on 5,008 chromosomes fall into two types of sub-networks (Figure 2). The majority of haplotypes (n = 80) belong to sub-networks consisting of haplotypes present in all human populations (“Cosmopolitan” haplotypes). The largest haplotype sub-network is centered on haplotypes found predominantly in Afrian populations, consistent with the “out-of-Africa” origin of modern human populations (Nielsen *et al.* 2017). The remaining haplotypes (n = 19) are present nearly exclusively in non-African populations, indicating an origin by intragression from archaic humans (“Archaic haplotypes”) (Figure 2).

### Expression of PVT1 exons 4A and 4B is upregulated in prostate cancer tissues from males of African ancestry

To determine the expression of PVT1 exons 4A and 4B in clinical specimens, samples were obtained from males who had undergone a prostatectomy or a transrectal ultrasound-guided biopsy. RNA extraction, cDNA synthesis, and real-time quantitative polymerase chain reaction (qPCR) were performed to assess the expression of PVT1 exons 4A and 4B.

**Figure 3:**
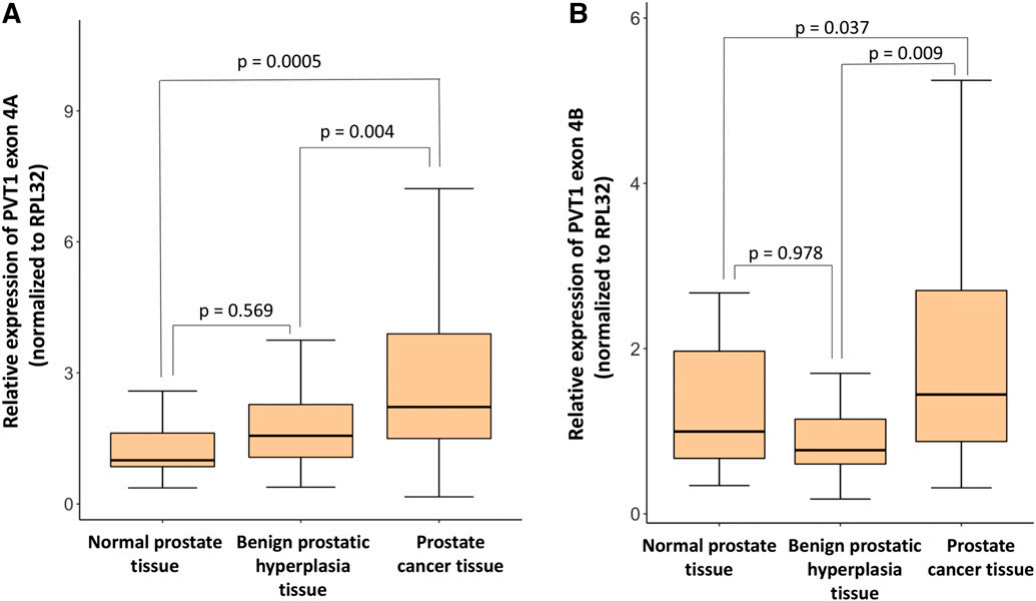
Expression of PVT1 exons 4A and 4B in normal, benign prostatic hyperplasia, and cancerous prostate tissue. (A) Expression of PVT1 exon 4A (B) Expression of PVT1 exon 4B. Data are presented as mean +/− standard error of the mean (SEM). The p values for statistical differences indicated here were determined from post-hoc tukey tests. All the criteria for significance were set at *P* < 0.05.

There is a statistically significant difference in the relative expression of PVT1 exons 4A (F(2,82) = 9.031, *P* = 0.0003) and 4B (F(2,82) = 5.294, *P* = 0.007) between groups as determined by one-way ANOVA (Figure 3A and 3B). Furthermore, post-hoc tukey tests show differences between relative mean expression of PVT1 exons 4A and 4B in prostate tumor tissues *vs.* normal prostate tissues: PVT1 exon 4A (prostate tumor: 3.15 ± 0.497, 95% CI [2.13, 4.17], benign prostate: 1.74 ± 0.163, 95% CI [1.41,2.07], and normal prostate: 1.28 ± 0.141, 95% CI [0.989, 1.57], *P* = 0.0005 for normal prostate tissue *vs.* prostate cancer tissue and *P* = 0.004 for benign prostatic hyperplasia *vs.* prostate cancer tissue); PVT1 exon 4B (prostate tumor: 2.217 ± 0.360, 95% CI[1.47, 2.95], benign prostate: 1.17 ± 0.176, 95% CI [0.821,1.53], and normal prostate: 1.25 ± 0.169, 95% CI [0.900, 1.60], *P* = 0.037 for normal prostate tissue *vs.* prostate cancer tissue and *P* = 0.009 for benign prostatic hyperplasia *vs.* prostate cancer tissue). However, no significant differences in expression are observed between expression by normal prostate and benign prostatic hyperplasia tissues either for PVT1 exon 4A (*P* = 0.569) or PVT1 exon 4B (*P* = 0.978). Paired *t*-test shows a significant difference in the expression profile for PVT1 exon 4B in prostate tumors with Gleason score ≥8 (2.98 ± 0.539, 95% CI [1.90,4.06]) as compared to those with Gleason score ≤7 (1.32 ± 0.340, 95% CI [0.645,2.07]) with *P* = 0.009. Further, PVT1 exon 4B overexpression appears to be very specific to PCa as there is no significant difference between PVT1 exon 4B expression between benign prostatic hyperplasia and normal prostate tissues.

### PVT1 exons 4A and 4B promote increased cell proliferation and migration

**Figure 4:**
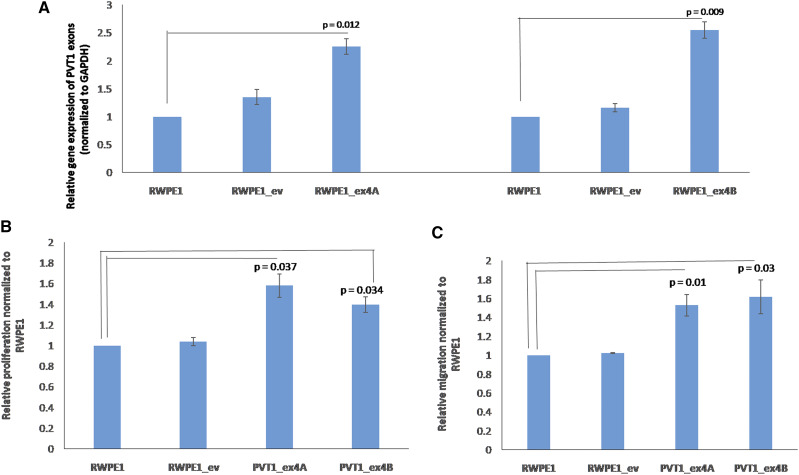
Stable overexpression of PVT1 exons 4A and 4B promotes proliferation and migration of prostate epithelial cells. (A) gene expression, (B) cell proliferation, (C) cell migration. Data are presented as mean +/− standard error of the mean (SEM). All the criteria for significance were set at *P* < 0.05. All experiments were done three different times. qPCR was performed in quadruplicates, using three different passages.

Plasmids containing PVT1 exons 4A and 4B were separately transfected into RWPE1, a non-tumorigenic prostate epithelial cell line. Effects of transient and stable overexpression of PVT1 exon 4A and 4B on cell proliferation and migration were assessed. As shown in Supplemental Figure 1, cells transiently overexpressing PVT1 exons 4A and 4B show significantly increased proliferative (Supplemental figure 1A) and migratory capacity (Supplemental figure 1B) in comparison to cells containing the empty vector, and untransfected RWPE1 cells. Furthermore, we successfully made stable cell lines overexpressing PVT1 exons 4A and 4B (RWPE1_ex4A and RWPE1_ex4B) and confirmed stable overexpression of both (Figure 4A) Notably, RWPE1_ex4A and RWPE1_4B are both significantly more proliferative and more migratory than RWPE1 with empty vector (RWPE1_ev), and non-transfected RWPE1 cells (Figure 4B and 4C).

## Discussion

GWAS help to identify genetic variations across the world and most interestingly, majority of cancer loci identified through GWAS locate to non-coding regions of the genome (Foley *et al.* 2017; Sud *et al.* 2017). These findings provide new avenues for investigation and demonstrate the usefulness of combining ancestrally diverse populations to discover risk loci for disease (Al Olama *et al.* 2014). For PCa, men of African ancestry are considered a ‘high risk’ population, with the highest incidence and mortality rates of any racial/ethnic population (Viale 2020). Nearly 4,500 Black males die from PCa annually (DeSantis *et al.* 2019). These long-standing racial/ethnic differences have yet to be explained. Association studies of PCa conducted in men of African ancestry have provided clear support for genetic differences in the allelic architecture of PCa across populations and strong support for a genetic basis underlying population differences in risk (Wasserman *et al.* 2010; Xiao *et al.* 2018).

The non-protein coding gene locus plasmacytoma variant translocation 1 (PVT1) is located at human chromosome 8q24 and is dysregulated in several cancers (Huppi *et al.* 2008). PVT1 gives rise to several alternatively spliced non-coding transcripts and microRNAs (Ling *et al.* 2015; Zhang *et al.* 2015; Zeng *et al.* 2015; Huang *et al.* 2016;; Iden *et al.* 2016; Lan *et al.* 2017; Chang *et al.* 2018; Xiao *et al.* 2018; Yu *et al.* 2018). There are at least twelve exons of PVT1, which are differentially expressed and may have distinct functions (Ling *et al.* 2015; Ilboudo *et al.* 2015). Aberrations of PVT1 are associated with multiple types of cancer, but the individual exons of PVT1 have not yet been fully characterized (Guo *et al.* 2017). In this study, we demonstrated population differentiation at the PVT1 gene locus and confirmed the clinical relevance of PVT1 exons 4A and 4B in PCa in men of African ancestry.

Using the most recent full-genome variability panel from the 1000 Genomes project, we identified a string of 75 SNPs in a 11-kb region spanning PVT1 exons 4A and 4B as consistently showing the highest level of genetic differentiation between African and non-African populations. To our knowledge, this is the first report of population-level study of the PVT1 gene. From our study, we provide evidence that transcripts from PVT1 exons 4A and 4B have clinical relevance in PCa. Through histopathological analysis, we confirmed that PCa tissues obtained from sub-Saharan African Black males significantly overexpressed PVT1 exons 4A and 4B in PCa tissues in comparison to benign prostatic hyperplasia and normal prostate tissues. Furthermore, PVT1 exon 4B overexpression is very specific for PCa, and it may distinguish between indolent and aggressive PCa.

Here, we report that both transient and stable overexpression of PVT1 exons 4A and 4B induce cell proliferation and migration. Notably, we have established a subline (RWPE1_ex4A and RWPE1_ex4B) of a non-tumorigenic prostate epithelial cell line (RWPE1) now stably overexpressing PVT1 exons 4A and 4B. Not surprisingly, stable overexpression of PVT1 exon 4A and PVT1 exon 4B in the sublines similarly resulted in increased proliferative and migratory capability by prostate epithelial cells. Consequently, we are reporting for the first time that alternatively spliced long non-coding transcripts from PVT1 exons 4A and 4B are significantly overexpressed in a cancer-specific manner in PCa. It remains to be investigated whether the elevated risk for PCa is associated with 4A/4B haplotypes absent in the African populations (*e.g.*, archaic alleles) or with 4A/4B haplotypes that are common in the African populations. Future experimental and population studies may further reveal PVT1 exon 4A/4B haplotypes associated with elevated risks of PCa in men of African ancestry.

In conclusion, our work has elucidated the important roles of PVT1 exons 4A and 4B in PCa. Consequently, transcripts from PVT1 exons 4A and 4B may have potential utility as diagnostic, prognostic, and therapeutic biomarkers in PCa.
